# Antigen Diversity in the Parasitic Bacterium *Anaplasma phagocytophilum* Arises from Selectively-Represented, Spatially Clustered Functional Pseudogenes

**DOI:** 10.1371/journal.pone.0008265

**Published:** 2009-12-15

**Authors:** Janet E. Foley, Nathan C. Nieto, Anthony Barbet, Patrick Foley

**Affiliations:** 1 Department of Medicine and Epidemiology, School of Veterinary Medicine, University of California, Davis, California, United States of America; 2 Department of Pathobiology, University of Florida, Gainesville, Florida, United States of America; 3 Department of Biological Sciences, California State University, Sacramento, California, United States of America; University of Missouri-Kansas City, United States of America

## Abstract

*Anaplasma phagocytophilum* is a tick-transmitted bacterial pathogen of humans and other animals, and is an obligate intracellular parasite. Throughout the course of infection, hosts acquire temporary resistance to granulocytic anaplasmosis as they develop immunity specific for the major antigen, major surface protein 2 (Msp2). However, the bacterium then utilizes a novel recombination mechanism shuffling functional pseudogenes sequentially into an expression cassette with conserved 5′ and 3′ ends, bypassing host immunity. Approximately 100 pseudogenes are present in the only fully sequenced human-origin HZ genome, representing the possibility for almost unlimited antigenic diversity. In the present study, we identified a select group of 20% of the *A. phagocytophilum* HZ *msp2* pseudogenes that have matched preferentially to human, canine, and equine expression cassettes. Pseudogenes cluster predominantly in one spatial run limited to a single genomic island in less than 50% of the genome but phylogenetically related pseudogenes are neither necessarily located in close proximity on the genome nor share similar percent identity with expression cassettes. Pseudogenes near the expression cassette (and the origin) are more likely to be expressed than those farther away. Taken together, these findings suggest that there may be natural selection pressure to retain pseudogenes in one cluster near the putative origin of replication, even though global recombination shuffles pseudogenes around the genome, separating pseudogenes that share genetic origins as well as those with similar identities.

## Introduction

α-Proteobacteria of the genus *Anaplasma* are obligately parasitic, intracellular, tick-transmitted pathogens of humans and other animals. *Anaplasma phagocytophilum* resides within neutrophils of numerous domestic animals, wildlife, and humans, and induces cytokine-mediated fever, malaise, myalgia, hematological and hepatic abnormalities, and occasionally central nervous system disease, susceptibility to secondary infection, organ failure, acute respiratory distress syndrome, and death variably in horses, humans, cattle, goats, sheep, and dogs [1]. In the United States, reservoir hosts include white-footed mice (*Peromyscus leucopus*), chipmunks (*Tamias* spp.), tree squirrels (*Sciurus* spp.), and possibly woodrats (*Neotoma fuscipes*)[2], [3], [4], [5]. Over the course of infection with *A. phagocytophilum*, hosts acquire temporary resistance to disease as they develop immunity specific for the major antigen, major surface protein 2 (Msp2). However, the bacterium then utilizes a novel recombination mechanism shuffling “functional pseudogenes” of the pfam01617 family (common within Anaplasmataceae) sequentially into an expression cassette with conserved 5′ and 3′ ends, bypassing host immunity and contributing in some host species to persistent infection [6]. In this case, pseudogenes, the equivalent of repeat DNA donor regions, are considered functional in that they serve as donor DNA into a functional expression cassette.

A hyper-recombination phenotype such as that expressed by *A. phagocytophilum* to generate antigenic variability may be an evolved response to complex ecologies in which there is intense selection (immune) pressure from its hosts [7]. The mechanism for the production of pseudogenes could be that remnant gene fragments occur as a side-effect of inaccurate duplication. However, the accumulation of pseudogenes represents a balance between their production, retention because of selection, and removal due to selection or drift [7]. There are costs to accumulating and using functional pseudogenes in recombination to avoid immunity [8]. Such costs could include the metabolic expense required by the bacteria to replicate potentially extraneous DNA and the danger of producing unfit progeny either through inaccurate recombination or through recombination yielding a suboptimal phenotype that does not successfully escape host immunity.

Patterns of pseudogene accumulation and utilization have been incompletely described for *Anaplasma* spp. Important differences exist between *A. phagocytophilum* and its closest relative, *A. marginale.* For the former species, more than a hundred functional pseudogenes have been found within a fully sequenced genome, while in contrast *A. marginale* contains only five to seven *msp2* pseudogenes [9], [10]. It is not clear in either species whether some of the pseudogenes are likely to be used (or have been used) more often than others, and whether there is any pattern to their spatial organization within the genome, either by being clustered in certain regions or by having some nearby genes or patterns of DNA in common with each other. In the present study, we evaluate the evolutionary history of functional pseudogenes within the only fully sequenced *A. phagocytophilum* genome. Specifically, we estimate the phylogeny of the *msp2* pseudogene family of the *A. phagocytophilum* HZ genome, investigate the spatial distribution of pseudogenes and whether there are spatial clusters on the chromosome where pseudogenes are over-represented, and evaluate the incorporation of pseudogenes into expression cassettes, based on genetic identities of pseudogenes with expressed *msp2* genes across 199 expression cassette sequences.

## Results

**Figure 1:**
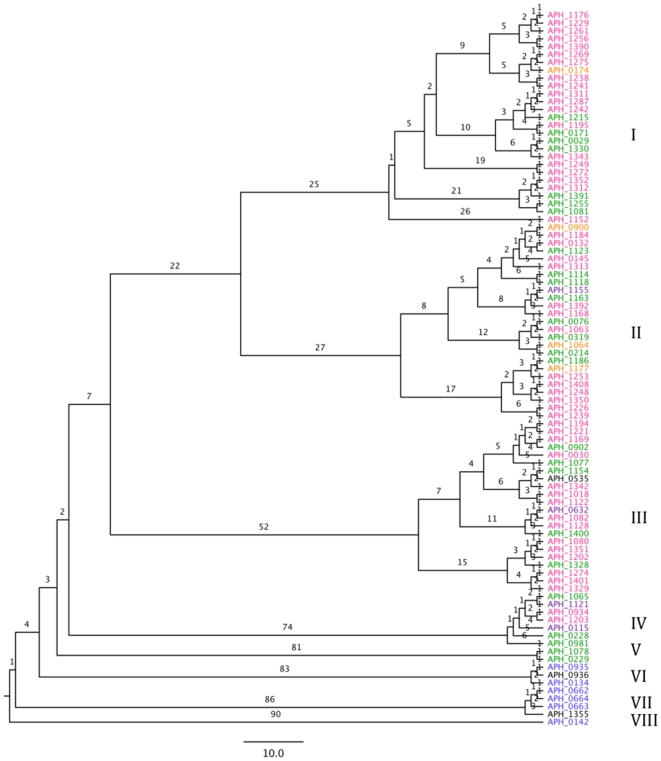
Unrooted maximum parsimony tree. Maximum parsimony tree representing relatedness of DNA from the *msp2* gene of 91 pseudogenes in the HZ strain of *Anaplasma phagocytophilum.* Numbers correspond to lengths of branches based on parsimony informative characters. Colors give maximum percent identity of each taxon with some *msp2* expression cassette, where black is ≤40% maximum identity, blue is 41–59%, green is 60–69%, purple is 70–79%, orange is 80–89%, and pink is ≥90% maximum identity.

Ninety-one *A. phagocytophilum* functional pseudogenes of the pfam01617 family were analyzed using maximum parsimony, yielding a tree that included eight major clades containing highly similar sister taxa but which were quite divergent from others, represented by many nucleotide changes at deeper nodes and longer branch lengths (Figure 1). The most parsimonious tree had a total branch length of 7662 with a mean sequence length of 793.9 bps (range = 192–1355 bps). The mean G+C content was 44.6% (A = 28.7%, C = 17.2%, G = 27.5%, T = 26.6%) compared to the G+C content of the entire *A. phagocytophilum* genome (41%). Following alignment with Prankster, 6452 characters were included in the parsimony analysis that included 4664 constant characters, 886 characters that were parsimony-uninformative, and 902 parsimony-informative characters.

**Figure 2:**
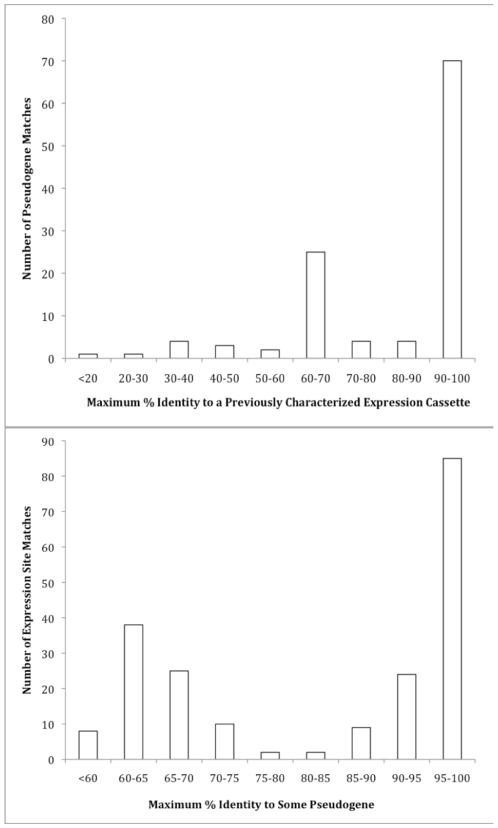
Maximum genetic identity . 2A. Frequencies of maximum genetic identity among 94 *A. phagocytophilum* HZ pseudogenes with any of the currently known *msp2* expressed alleles found in isolates from diverse hosts. 2B. Maximum genetic identity. Frequencies of maximum genetic identity among 199 sequenced *msp2* expression cassette with any of 94 *A. phagocytophilum* HZ pseudogenes.

Based on calculation of protein identity, 44 of 94 pseudogenes (46.8%) were almost identical (approximately 100%) to at least one expression cassette, while 26/94 (27.7%) had high identity (≥90%) (Figure 2a). However, maximum identities varied considerably, ranging from 16.8 to 100%. There were two peaks in this representation of pseudogene identities, one above 90% identical to an expressed gene and a second peak from 60–70% identities. The mean maximum identity across all pseudogenes was 80.4% (21.4 s.d.), and the mode was 100%. Of the 46 pseudogenes with high identity to some expression cassette, in almost all cases, there was only high identity to a single cassette. Pseudogenes with moderate (50–87%) vs. high (>90%) identity to some expression cassette were compared with respect to the host to which they matched most highly by chi-square statistical tests (Table 1). Moderately matched pseudogenes disproportionately included those matching most highly to deer-origin (strain Ap-1) and European-origin sources, while highly matching pseudogenes were more likely matched to humans, horses, and dogs (P = 0.001).

**Table 1:** Pseudogene identity with host type.

Strain	Ap-1	Human-origin	Equine-origin	Canid- origin	European (diverse hosts)	Wild rodent-origin
High identity	11 (22%)	5 (10%)	12 (24%)	7 (14%)	13 (26%)	1 (2%)
Low identity	11 (30%)	3 (8%)	6 (16%)	4 (10%)	12 (32%)	1 (0%)

Numbers (and percentages) of pseudogenes with maximum identity to an expression cassette from six different host categories, divided into high maximum identity (90% or greater) compared with moderate to low (50–87%).

The transposed summary comparing number of expression cassettes that matched to each pseudogene was bimodal (Figure 2b). Most (109, 54.8%) expression cassettes matched highly (≥90%) to at least one pseudogene, while a second peak from 60–70% identity included 63 cassettes (31.7%). The identities ranged from 57.6–100%, with a mean of 84.0% (16.1 s.d.) and a mode of 100%.

There was some clustering on the parsimony tree of pseudogenes that had high percentage identity with expression cassettes (Figure 1). The largest clades with all highly identical terminal branches were clades I and III. In clade I, 19 of 27 (70%) taxa had from 90–100% identity to some expression cassette, with 14 of these matching to cassettes from either North American horses, carnivores, or humans; none of the taxa in this clade had less than 60% identity. For clades II and III, respectively 13 of 25 (52%) and 15 of 22 (68%) of taxa were highly identical to an expression cassette, although in clades II and III, several taxa had very low identities. Four clades contained all taxa with low or not-used identities, each of which represented sister-groups to the rest of the tree.

**Figure 3:**
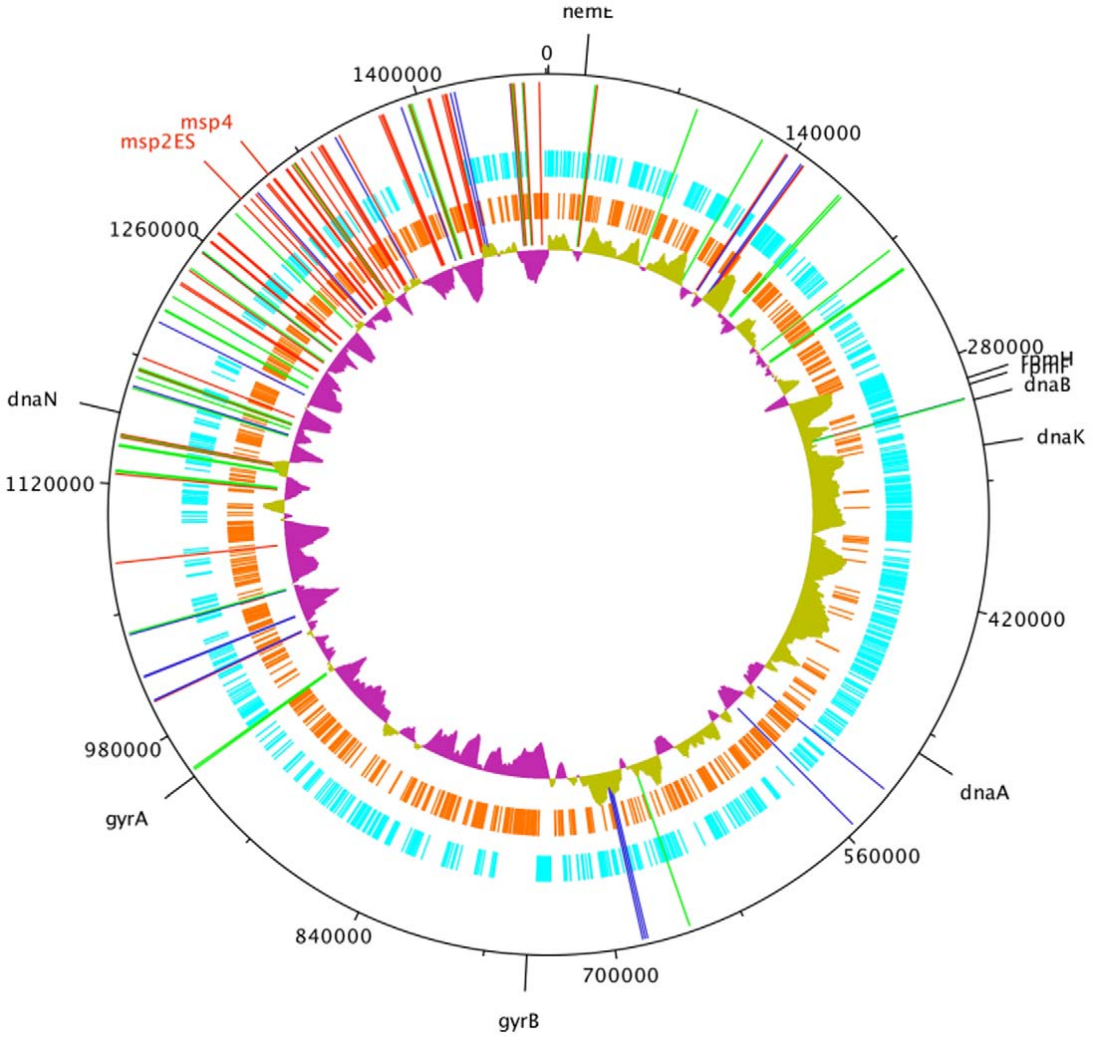
HZ pseudogenes on genome map. A genome map of all 113 predicted *msp2* pseudogenes of the *A. phagocytophilum* strain HZ. The most inner circle represents the guanine/cytosine skew, blue circle represents genes in the 3′ direction, orange circle represents genes in the 5 direction, and long bars represent the position of each *msp2* pseudogene. Red pseudogene bars correspond to 90–100% identity with some expression cassette, green bars to 60–90% identity, and dark blue bars to identities <60%. For orientation, the position of genes commonly associated with the origin of replication (*rpmH, rpmF, dnaA, dnaB, dna K, dnaN, gyrA, gyrB*) are included as well as the position of the Msp2 and Msp4 expression cassette (msp2ES).

Spatial and statistical analysis of the *A. phagocytophilum* HZ pseudogenes revealed two statistically significant (P = 2.2×10^−16^) clusters of pseudogenes, one from positions 26311–227059 bp and the other from 957413–1462420 bp, both bracketing the hypothesized origin of replication (Figure 3). There were six isolated pseudogenes at positions 303903–684696 located outside of the clusters. Combining clusters 1 and 2, all except six isolated pseudogenes were present in only 50% of the length of the genome. There were 25 short runs of two-five tandemly arranged pseudogenes where pseudogenes were either directly adjacent to each other or within one gene of the next pseudogene. These short runs almost never clustered together on the parsimony tree except for genes Aph_662, 663, and 664; and 935 and 936. Both of these short runs had low or essentially no identity with any yet sequenced expression cassette. Of the 25 runs, 8 (32%) showed concordance in identity with expression cassettes, with one having members all with low identity, three having members from 60–70% identity, and four having members all with high identity.

**Figure 4:**
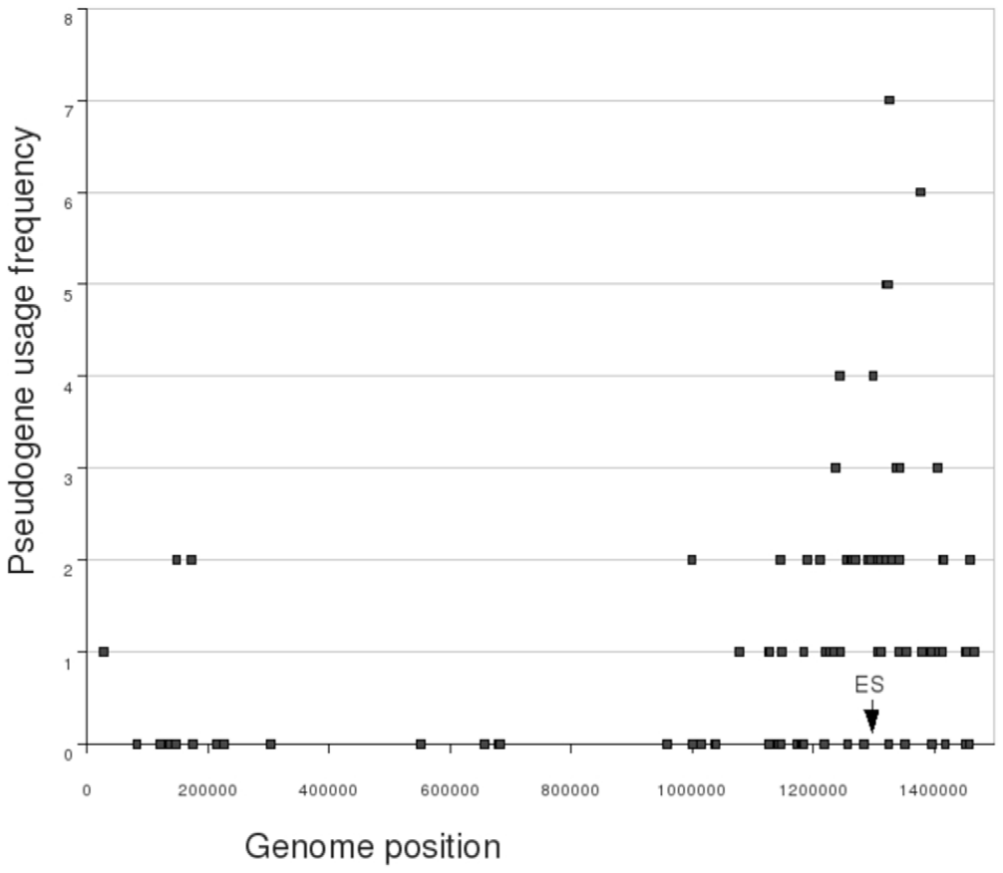
Position of highly used pseudogenes. Genome position and usage frequency of pseudogenes in the *A. phagocytophilum* HZ genome. Usage frequency refers to the number of expressed genes that have 90% or higher similarity to a given pseudogene. “ES” indicates the *msp2* expression site.

When the positions on the genome were compared between those pseudogenes with high identities to expression cassettes compared to those with low percent identities, the six non-clustered pseudogenes had a mean of 52.8% identity, compared with a mean of 72.2% for the first cluster and 84.1% for the second cluster. These differences were statistically significantly different (P = 0.009). Importantly, statistical analysis suggested that an individual pseudogene was more likely to be a sequence donor if it was closer to the *msp2* expression site (Figure 4). These pseudogenes near the expression cassette and origin were represented in 1–7 expressed genes (i.e. had greater than or equal to 90% similarity to the expressed genes), but more distant pseudogenes only were used from 1–2 times (Figure 4). Using ranked correlation, genomic distance from the expression cassette accounted for 21% of the variance in the ranking of maximum identity to some expression cassette (P = 2.4×10^−6^). There was no discernable pattern to how pseudogenes clustered on the parsimony tree relative to their positions on the genome.

## Discussion

In this study, we identify a select group of *A. phagocytophilum* HZ *msp2* functional pseudogenes that have matched preferentially to human, canine, and equine expression cassettes; show that pseudogenes cluster predominantly in one spatial run near the origin and expression cassette; and find that genetically related pseudogenes are not necessarily located in close proximity on the genome but may share similar percent identity with expression cassettes. Taken together, these findings are consistent with natural selection acting to retain in one cluster in the HZ genome specific pseudogenes with high identities to *msp2* cassettes from *A. phagocytophilum* strains from select hosts (horses, dogs, and humans). This could occur even though recombination shuffles pseudogenes in the genome, separating pseudogenes that share genetic origins.

Central questions surrounding major surface proteins in the *Anaplasma* genus are to what degree different species utilize available genetic variability to evade host immunity and why *A. phagocytophilum* maintains an order of magnitude higher number of functional pseudogenes compared with *A. marginale* (and in fact most other rickettsias). Both species have relatively small genomes (1.2–1.5 MB) and have single *msp2* expression cassettes that encode polycistronic mRNA [6], [11]. The majority of changes produced by gene conversion are in a central hypervariable region of *msp2* of about 280 bp. Variant Msp2 proteins are expressed during periods of bacteremia in association with production of serum immunoglobulins and serve to evade host immunity [12], [13], [14]. There are diverse *msp2* variants across naturally-infected animals, including humans in the U.S., wildlife reservoir species from the U.S., and sheep and dogs in Europe [6]. Thus pseudogenes serve an important role in protecting *Anaplasma* species of bacteria from the host, yet it was not clear how they originate, how they are related, and whether some are used differentially in expression.

Pseudogenes arise in the *A. phagocytophilum* genome by mechanisms that are not well understood and function by being shuffled into an expression cassette using the nonreciprocal RecF pathway [15]. Other organisms use related mechanisms of pseudogene incorporation into expression sites [16]. For the *Borrelia hermsii* variable major protein (vmp), there is duplicative unidirectional, nonreciprocal transfer of nucleotides from a donor gene to create a new expression site downstream of a promoter. A second mechanism is reconstitution of a pseudogene to a functional expression site by homologous recombination to juxtapose fragments of *vmp7* with the pseudogene, making it a full gene [17]. Other prokaryotic examples of pseudogene incorporation into variable antigens occur in the agents of gonorrhea (*Neisseria gonorrhoeae*) and Lyme disease (*B. burgdorferi*), which undergoes extensive gene conversion through recombination between the *vlsE* expression site and any of 15 adjacent silent *vls* cassettes located on a linear plasmid [18]. Important in all these systems is what determines the frequency of usage of individual sequence donors for recombination into an expression site. In *B. hermsii*, usage frequency is related to the degree of homology between extragenic sequence elements in the donor locus and similar regions in the recipient expression locus [19]. In African trypanosomes, it may be related to the probability of activation of donor gene sequence from structurally different genomic loci, e.g. from minichromosomes or tandem gene arrays in larger chromosomes [20]. Evidence presented in this study suggests that in *A. phagocytophilum*, usage frequency may be determined by genomic distance of the pseudogene from the expression locus.

Once pseudogenes are created, they may be retained in the bacterial genome if there is positive selection on them. For example, in *Wolbachia* which is a related member of the family Anaplasmataceae, the major surface protein contains four hypervariable regions, with recombination shuffling DNA to create a mosaic gene [21]. Although purifying selection operates to minimize variability, the hypervariable regions show evidence of strong directional selection, possibly consistent with selection applied to the *msp2* of *A. phagocytophilum*. In general, bacteria have consistently small genomes based on an interplay of two evolutionary forces: deletional bias and drift to remove unnecessary DNA opposed by selection on genes (or functional pseudogenes) to retain useful DNA [22]. Smaller genomes derive from larger genomes [23]. Intracellular bacteria tend to evolve distinctly smaller genomes than extracellular ancestors, presumably reflecting selective benefits for fewer genes as unnecessary environmental but also DNA repair and replication genes are deleted.

The present study analyzes patterns of functional pseudogenes in the fully sequenced HZ genome. Availability of additional fully sequenced genomes will enhance our understanding of mechanisms of pseudogene evolution, but several patterns may be identified at least preliminarily from the HZ genome. Patterns detected are consistent with positive selection to retain functional pseudogenes in a restricted area of the genome, selection to remove some mutated or degraded pseudogenes, and underlying roles of recombination and drift assorting and removing pseudogenes after they are created. An alternative explanation could be increased local duplication but this would not account for the observation that pseudogenes most closely related phylogenetically were not necessarily positioned proximally in the genome. Rather, phylogenetic analysis showed that closely positioned taxa on the genome were not adjacent on the tree, suggesting recombination to move pseudogenes around once produced. There were several clades in which all members had similar identities with expression cassettes with others all containing essentially unmatched pseudogenes. Interestingly in at least one clade, even though pseudogenes were closely related phylogenetically and were not positioned near each other on the genome, they did match to a very high degree with expression cassettes from dogs, horses, and humans. The finding that most of the pseudogenes had 90–100% identity with some expression cassette, over-representing cassettes from humans, horses, and dogs, has two key implications. First is that these pseudogene variable regions were essentially completely incorporated into a cassette, as opposed to a mosaic product of segmental gene conversion. The latter product of segmental gene conversion has been reported for *A. marginale* but in low frequency, only after host-imposed immune pressure, and with an important fitness disadvantage in the absence of immunity, compared with simple recombinants of whole hypervariable region domains [11], [24]. Secondly, the match to humans, horses, and dogs possibly reflects the source of the complete sequenced genome, i.e. a human-origin strain and a bias toward sampling acute infections in these species. It is interesting that within the “high-matching” species, some of the lowest matches are to humans, possibly consistent with a more recent exploitation of this niche or host. It is possible that matches will be higher for European cassettes for functional pseudogenes in a homologous European genome or Ap-1 cassettes if the genome originated from a deer strain. This is consistent with recent data from Japan. Analysis of *msp2* genes from *I. ovatus* and *I. persulcatus* showed high diversity in *I. persulcatus-*derived strains but less so for *I. ovatus,* while all tick strains occurred as a single clade, with a sister clade consisting of strains from deer [25]. Alternatively, some cassettes may be inherently more fit, which is consitent with the data from *A. marginale*, where the frequency of use of *msp2* alleles was non-random with, as an example one allele present in 29% of all produced variants [24].

There were some pseudogenes with low to negligible matches to any reported cassette. This could be because we have obviously not sampled all expression cassettes that have occurred in nature or because these pseudogenes are mutated, degraded versions of functional pseudogenes. Although 94 pseudogenes were included in this analysis, the original published genome reported 113. Of these, all show some homology with the pf01617 gene family, but many of the predicted proteins lacked characteristic LAKT residues and conserved 5′ features that facilitated alignment or other sequence features that would allow them to contribute to variable antigen expression. These low identity and degraded pseudogenes appear not to be functional for antigen variation and likely will be removed from the genome by drift and selection.

The spatial distribution of pseudogenes is non-random, with numerous runs of 2–5 pseudogenes in tandem, almost all lying within only half of the genome, and more expressed pseudogenes lying near the HZ expression cassette than expected by chance. It may be that the presence of a pseudogene or some other local feature creates a template hotspot facilitating hypermutability, duplication, and gene conversion. Alternatively, globally distributed inversions could operate to promote reorganization of pseudogenes, but those not retained within the cluster are removed by selection. Clustering does occur for *A. marginale* pseudogenes as well, although there are far fewer of them than in *A. phagocytophilum* and *A. marginale* pseudogenes are not preferentially recombined as a function of their proximity to either the origin or the expression cassette, in contrast to the data presented here. However, some of the difference may be due to the statistical power for *A. marginale:* the presence of fewer pseudogenes could make it more difficult to detect clustering even if it occurred. In general, unlike in many other bacteria, rickettsial genes, including those responsible for genomic organization and replication, exist dispersed in the genome as a result of frequent recombination, making the clusters of functional pseudogenes that much more unusual [26]. This suggests that natural selection could operate to retain a cluster of functional pseudogenes in one location. The localized repeats could suggest local gene duplication reminiscent of the tandem placement of pseudogenes for surface antigens in *Ehrlichia* spp. [27]. However, it is interesting in the present study that very few of the members of short runs were in the same clades. It may be consistent, however, with the previously suggested hypothesis that localized gene duplication removes constraints on the duplicated gene and is rapidly followed by mutation and recombination [28].

The nature of the selection pressure for *A. phagocytophilum* pseudogenes varies across hosts. It is unlikely that extensive selection occurs on the pseudogenes during residence in the tick. *Ixodes* spp. ticks transmit the infection and hard ticks only feed once per stage, thus ticks must acquire the infection as larvae or nymphs and transmit infection in the subsequent stage (as nymphs or adults). Infection does not appear to cause injury to ticks and ticks do not attenuate infection. In vertebrate hosts, acute, self-limiting infection is typical for some hosts: despite the ability of *A. phagocytophilum* to vary antigens, host immunity succeeds in containing infection within 2–4 weeks in horses, humans, and cattle. One study showed the emergence of variant Msp2 antigens over the course of infection in horses, nevertheless horses limited the infection to under a month [14]. Hosts for which chronic infection has been described include rodent reservoirs such as woodrats and chipmunks, as well as sheep and dogs in some cases [4], [29], [30], [31], [32]. Because infection-limiting hosts seemingly impose strong negative selection on *A. phagocytophilum*, there is strong positive selection for pseudogenes to contribute to immune evasion, which could partly account for the benefit of having numerous functional pseudogenes. However, the potential for combinatorial diversity with so many pseudogenes is vast, and the available pseudogenes in *A. phagocytophilum* far exceed those in *A. marginale*. It may be that the large number of pseudogenes in *A. phagocytophilum* is driven partly by the complex ecology in which this pathogen exists, with so many diverse hosts and differing selection pressures. Evolutionary analysis has suggested that *A. marginale* and *A. phagocytophilum* split into separate species 43,000,000–78,000,000 years ago and that *A. marginale* is basal [33]. Thus, fewer pseudogenes could be the ancestral character, and occur in a bacterium that is host-specialized and only infects cervids and bovids. This would support the notion that ecological complexity of *A. phagocytophilum*, with such a diverse array of chronic and infection-limiting hosts, could be an important factor determining the number of pseudogenes retained in the genome.

A complex interplay of duplication, recombination, drift, mutation, and selection has created a landscape of more than one-hundred functional and degraded pseudogenes in the *A. phagocytophilum* HZ genome. Pseudogenes cluster in space but closely positioned pseudogenes may not be closely related. There is a large group of pseudogenes that match closely with cassettes disproportionally arising from US dogs, humans, and horses and pseudogene usage appears related to distance from the expression site. An important next step to this analysis will be comparison of our findings with patterns from other strains as additional *A. phagocytophilum* genomes are sequenced, particularly from Old World strains and reservoir hosts. Additionally, the placement of these results into the genus-wide context by comparing *A. phagocytophilum* with *A. marginale* will help clarify how pseudogenes evolve and solve the puzzle as to why an obligately parasitic rickettsia would maintain such a large repertoire of functional pseudogenes as seen in the *A. phagocytophilum* HZ genome.

## Materials and Methods

Ninety-four *msp2* pseudogene DNA sequences were obtained for the New York state, US human-origin strain *A. phagocytophilum* HZ (NC 007797)[9]. Ninety-one of these sequences could be aligned using Prankster (http://www.ebi.ac.uk/goldman-srv/prank/prankster/) using the Hasegawa-Kishino-Yano (HKY85) substitution model and program defaults. Phylogenetic analysis was conducted using the program PAUP* [34] to construct a parsimony tree with default values. Gaps were considered missing. The unrooted tree was edited and displayed using FigTree v1.2 (http://tree.bio.ed.ac.uk/software/figtree/).

To determine the 1∶1 amino acid sequence identities between the pseudogenes and expression cassette sequences, 199 complete *A. phagocytophilum msp2* expression site gene sequences were included in the study. The source of these sequences including host origin are described in three earlier publications [6], [13], [35]. (Although more expression cassette sequences were available, only one per animal was used for the analysis in this paper). Expression cassettes and the 94 HZ pseudogenes were translated to amino acids, and then a Fasta file was analyzed with MatGat V2.02 (Matrix Global alignment tool)[36] using the BLOSUM 50 model. These 94 pseudogenes were chosen because they contained 5′ or 3′ conserved regions and a LAKT motif that allowed for analysis. The percentage identities determined by MatGat for an all-against-all comparison were exported to a Microsoft (Redmond, WA) Excel spreadsheet. Summary statistics of percent identities were obtained in the program R (R-Development Core Team, http://www.r-project.org). The maximum identity for each pseudogene against all possible expression cassettes was determined and the mean, standard deviation, mode, and range of maximum identities calculated. These statistics give the likelihood that any particular pseudogene was used in any expression. Maximum identities were discretized for analysis as nearly perfect (99–100% identity between a pseudogene and an expression cassette), high identity (90–98%), moderate (70–89%), low moderate (60–69%), low (40–59%), and not used (<40%). A transposed summary also was created to evaluate the maximum identity of all pseudogenes against each given expression cassette, to capture the likelihood that any given cassette could have sampled from particular vs. multiple pseudogenes. In order to determine whether pseudogenes that had high or nearly perfect identity with expression cassettes were more likely to match with particular types of hosts compared with pseudogenes with moderate and low moderate identities, a chi-square contingency test was performed with the following hosts: deer, human, horse, carnivore, European animals, and woodrats.

For spatial analysis of the distribution of pseudogenes on the two genomes, we used a Wald-Wolfowitz runs test in the R package “lawstat” with units in the analysis = genes. An ANOVA test was used to compare mean percent identities of pseudogenes with expression cassettes in the three identified spatial clusters on the genome. A Spearman rank correlation coefficient was calculated in order to assess whether maximum identities of pseudogenes near the expression site were higher than for those more distantly positioned pseudogenes. For all tests, a value of P≤0.05 was considered evidence of statistical significance.
